# Knowledge, Attitudes, and Behavior in Avoiding Secondhand Smoke Exposure Among Non-Smoking Employed Women with Higher Education in Jordan

**DOI:** 10.3390/ijerph8114207

**Published:** 2011-11-09

**Authors:** Huda Gharaibeh, Linda Haddad, Sukaina Alzyoud, Omar El-Shahawy, Nesrin Abu Baker, Mary Umlauf

**Affiliations:** 1 School of Nursing, Jordan University of Science and Technology, P.O. Box 3030, Irbid, Postal Code 22110, Jordan; E-Mails: hudag@just.edu.jo (H.G.); nesrin@just.edu.jo (N.A.B.); 2 Institute for Drug and Alcohol Studies, School of Nursing, Virginia Commonwealth University, Richmond, VA 23298, USA; 3 School of Nursing, Hashemite University, Amman, Postal Code 13133, Jordan; E-Mail: sukaina-alzyoud@hu.edu.jo; 4 General Medical Management, Ain Shams University, Cairo, Postal Code 11355, Egypt; E-Mail: oshahawy@gmail.com; 5 Institute of Drug and Alcohol Studies, Virginia Commonwealth University, Richmond, VA 23298, USA, E-Mail: elshahawyo@vcu.edu; 6 School of Nursing, University of Alabama, Tuscaloosa, AL 35487, USA; E-Mail: mgumlauf@ua.edu

**Keywords:** secondhand smoke, Jordan, higher education, women, socioeconomic status

## Abstract

Secondhand smoke (SHS) exposure is a serious public health threat worldwide; in the developing world there are less serious efforts towards controlling women’s and children’s exposure to SHS. Knowledge, attitudes and avoidance practices among Jordanian women have never been thoroughly studied. The purpose of this study was to assess the knowledge, attitudes, and avoidance behavior towards SHS exposure among employed Jordanian women with higher education.

**Methods:**

A survey was conducted among employed Jordanian women at two universities. A total of 209 women were included in the analysis. Two questionnaires regarding SHS exposure were used to measure knowledge, attitudes and avoidance practices.

**Results:**

Most respondents were regularly exposed to SHS in various locations during daily life, even though they were very knowledgeable about the dangers of SHS exposure for women and children. However, the subject’s attitudes and avoidance behavior did not reflect the level of knowledge about SHS risks. The results suggests there is a large discrepancy between SHS exposure, knowledge, attitudes and avoidance behavior among highly educated Jordanian women that is likely influenced by culture and traditional gender roles. Public health initiatives are needed in Jordan to address public policy, institutional practices and to empowerment of women to reduce SHS exposure.

## 1. Introduction

Tobacco is the leading preventable cause of morbidity and mortality in developing countries and is expected to remain so through 2020 [1,2]. There are no safe levels of exposure to smoke for non-smokers [3]. In developed countries there have been major efforts to promote smoking cessation and to educate the public about the adverse effect of smoking, as well as the hazards of SHS. Conversely, most developing countries have not delivered or promoted educational efforts about smoking risks or SHS exposure. There are a limited number of studies that have examined or measured parental knowledge or beliefs regarding the adverse health effects of SHS exposure. These studies found that parents typically have a basic or less comprehensive understanding of the harm inflicted by SHS on pregnant women and children. These studies were done in different populations in developing countries across the globe; one of them was done in the Middle East [4,5]. Knowledge and attitudes regarding SHS exposure are much lower among people with lower socio-economic status (SES) [4]. Many other factors play a role in smoking and SHS exposure in developing countries, especially Arab countries that exhibit higher smoking rates. These factors are not fully understood.

Jordan is situated between Saudi Arabia, Iraq, Israel, and Syria. Thus it is a typical Middle Eastern country that represents a conservative culture, special social norms and a male dominated society. Officially, Jordan prohibits smoking in public places and workplaces. However, the enforcement of anti-smoking laws in most public locations is extremely poor [6]. This could be attributed to the fact that more than 50% of Jordanian men smoke. This indicates a substantial lack of correct knowledge and healthy attitudes towards SHS exposure within the general population. As such, smoking may be viewed as a social norm to the Jordanian society. Thus, curbing the tobacco epidemic will require efforts to de-normalize as a major effort of public health initiatives [4].

Among the few recent studies of SHS exposure among Jordanian women, Madanat *et al.* [7] conducted a random household survey to examine the knowledge about indoor air quality relating to respiratory illness and home environments among Jordanian women. The study used a self-report questionnaire and included two questions regarding SHS exposure. The results showed that even with knowledge of how indoor air quality contributes to respiratory symptoms as a moderator, most households (71%) included indoor smokers. However, the severity and quantity of SHS exposure was not measured in this study and neither was either attitude or avoidance behavior toward SHS exposure. Badran *et al.* [8] conducted a cross sectional study in the capital city Amman employing a convenience sample of 220 infants and mothers attending an outpatient pediatric clinic. This study identified SHS exposure using two measures: the mother’s self report of household exposure and cotinine levels from both infants (urine) and mothers (saliva). Mothers reported that 60% of the infants were exposed to SHS. Correspondingly, 36.4% of the infants had detectable levels (7.1 ng/mg) of urine cotinine. The study also found cotinine in saliva among eight of the 20 mothers with neonates (1–2 days old), implying *in-utero* SHS exposure. Abu Baker *et al.* [9] interviewed 300 postpartum women and reviewed their neonates’ health records for the purpose of measuring SHS exposure during pregnancy. The results showed that SHS exposure occurred primarily in the home, and the rate of hourly exposure was high across all three trimesters. Among women who reported SHS exposure at home, 52.7% reported their spouses as the smoker in question. Further, the average number of hours of SHS exposure per week in all settings (home, work, and outdoors) in the second trimester explained 22% of the variance in neonatal birth weight in the sample.

Although fewer women smoke in Jordan than men, women are exposed to high rates of SHS in the household setting. In this authoritarian culture and male dominated family structure, apparently men who are smokers rule many Jordanian families. In a typical Jordanian household, there are distinct gender roles where men take precedence over women in shaping the social practices at home [5,10]. Thus, empowering women could facilitate reduction of SHS exposure at the household level and the adverse effects among their children.

This study focused on working women with higher education profiles because SHS exposure was hypothesized to be the lower among this group to compare with other studies that reflect high SHS exposure rates among uneducated and less educated women in Jordan. The study setting was a university environment where workplace smoking should be limited due to institutional policy. Approaching this socio-economic group is the first such attempt in Jordan; the findings will serve as foundational data on women’s perceptions about SHS exposure to assist in developing tobacco control programs aimed at improving maternal-children health. The specific aim of this study is to assess the women knowledge, attitudes, and avoidance behavior towards SHS exposure among working women with higher education.

## 2. Methods

### 2.1. Setting and Population

Data were collected using a convenience sample of women employed at one of two large national universities (Jordan University of Science and Technology located in the northern area of Jordan and Hashemite University located more centrally in Jordan). The two universities employ around 1,500 women in staff and academic positions, and enroll more than 40 thousand students in many educational disciplines.

### 2.2. Data Collection Protocol

This study was approved as exempt level study by the institutional review board of Jordan University of Science and Technology. During a three months period, from August to October 2010, the investigators and research assistants collected the data. They distributed a flyer and letters across the two universities to recruit women employees. The flyer and the letter explained the purpose of the study, and invited women who were non-smokers or had not smoked for the prior year to volunteer for the study. After this flyer distribution, the researcher distributed survey packets to 302 adult female employees. The survey packet included a cover letter, a demographic data sheet and two questionnaires along with a return envelope. As described in the cover letter, investigators were available by phone and email to answer questions and provide clarification about the study during the interval of data collection. The recruited women were informed that study participation was voluntary and responses would be anonymous. Of those who were given surveys, 211 women returned the questionnaires to yield a response rate of 69.9%. Of those, 209 surveys were fully complete and subsequently used for data analysis. Although this sample is not representative of the female Jordanian population at large, knowledge and attitudes towards SHS exposure were hypothesized to be higher in this group than in women with lesser educational backgrounds [11].

### 2.3. Instruments

The SHS data was collected using two questionnaires:

#### Household Secondhand Smoke Exposure Questionnaire

This measured SHS exposure sites (home, work, elsewhere). The questionnaire was adapted from surveys by Wipflie [12] and Glasgow *et al.* [13] and translated into Arabic. The questionnaire had four sections: (1) general information and demographic characteristics; (2) smoking behavior; (3) exposure to SHS; (4) attitudes towards the SHS control policies which consists of five questions with responses on a 4-point scale (1 = disagree; 2 = somewhat disagree; 3 = somewhat agree; 4 = agree). Cumulated scores were summed up at a range of from 9 to 45, with higher scores indicating a better behavior to avoid SHS level.

#### Knowledge, Attitudes, and Preventive Efforts to Avoid SHS Exposure Scale

This questionnaire covered three domains: (1) knowledge of the adverse effects associated with SHS exposure; (2) attitudes and personal feelings toward SHS exposure; (3) avoidance or preventive efforts undertaken by the women when exposed to SHS in their immediate environment. The questionnaire consists of two subscales. First, the Knowledge and Attitude Subscale developed by Kurtz *et al.* [14] that includes 6 items asking women about their knowledge and six items measures the participants attitudes toward SHS exposure. The subscale is in the form of 5-point Likert-type response scale (1 = strongly agree; 2 = agree; 3 = undecided; 4 = disagree; 5 = strongly disagree). Cronbach’s alpha coefficient of this scale was 0.79. Second, the Avoidance of SHS Exposure Subscale that was developed by Martinelli [15]. This subscale included nineteen items that assessed the participants’ efforts to prevent SHS exposure. This subscale is in the form of 4-point Likert-type response scale (1 = almost always true; 2 = usually true, 3 = usually not true; 4 = almost never true). Cumulative scores were summed and yielded a range of 19–76 with higher scores indicating a greater avoidance of SHS. Cronbach’s alpha coefficient of this scale was 0.82. Two bilingual qualified faculty members at the University of Science and Technology in Jordan translated each scale into Arabic. Both translators are native Arabic speakers who had previous experience in conducting tobacco survey research. Only the Arabic versions were used in this study.

### 2.4. Data Analysis

The Statistical Package for the Social Science (SPSS) version 17 was used for data analysis. The alpha level was set at 0.05 to determine statistical significance. Descriptive analysis were conducted to characterize self reported SHS exposure, worksite smoking policy, husband’s smoking status, knowledge and attitudes toward SHS exposure, and SHS avoidance practices. Chi-square tests were performed to estimate the bivariate difference between anti-smoking workplace policies, husband smoking and avoidance of SHS exposure by subjects. Avoidance behaviors were also compared between subjects in clerical positions and clerical administrator positions versus those in academic positions using the Analysis of Variance.

## 3. Results

### 3.1. Sample Characteristics

Participants’ age ranged between 28 and 58 years old with a mean of 34.6 years old (SD ± 7.2). The results showed that 82.3% were working in clerical administrative professional jobs, while 17.7% were working in academic positions. All participants had a college graduate degree or higher. All participants were married and living with their husbands in the same house. As mentioned in the sample’s selection criteria, none of the subjects were smokers or had smoked within the prior year. The majority of participants (67.5%) spent most of their time in an indoor environment.

### 3.2. Worksite Smoking Policies

Despite the fact that workplace anti-smoking policies existed at the time of the survey, when participants were asked about the presence such policies only 52.2% reported being aware of these policies. Nearly one-third (32.1%) did not think that their university had anti-smoking policies, and 15.8% were not aware whether such policies existed. Furthermore, when asked about details of existing indoor smoking policies, 54.5% reported restrictions in all closed indoor areas, while 22% reported that smoking was allowed in some closed areas, and 5.7% reported that indoor smoking was allowed in all areas. Moreover, 17.7% reported not knowing anything related to indoor smoking policies, this is despite their existence when the study was conducted.

### 3.3. Husband Smoking

More than half (55%) of participants reported living with a smoking husband; the average number of cigarettes smoked by the husbands was 23.2 (SD ± 11.3) per day. More than one-third (367%) of husbands smoked inside their homes; and many (24.9%) subjects reported that other family members also smoked in the home. In total, almost two thirds (59.6%) of subjects reported that someone (husband or other family member) smoked inside the house. When asked to estimate daily SHS exposure inside and outside their homes, subjects reported a mean of 5.5 (SD ± 5.5) hours per day. Additionally, subjects reported SHS exposure for an average of 6 (SD = 6.2) days per week in any setting.

### 3.4. Self Reported Locations of SHS Exposure

**Figure 1:**
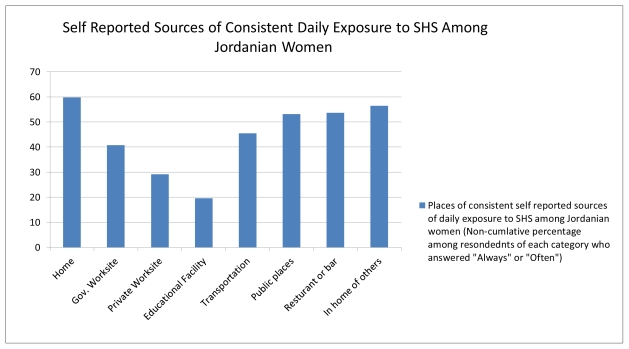
Self-reported sources of consistent daily exposure to SHS (N = 209).

Women in the study were asked to identify locations of SHS exposure. The results presented in Figure 1 show the non-cumulative percentage of women who reported SHS exposure (response of “Always” or “Other”) in the following settings: home, government worksite, private worksite, educational facility or school, transportation means, public places, restaurants or bars and homes of others. Exposure to SHS in their own homes or the homes of others was slightly higher than exposure rates at restaurants and other public places.

### 3.5. Knowledge and Attitudes about SHS Exposure

Almost all women in the study (97.6%) perceived SHS exposure as dangerous to the health of the exposed non-smoker (Table 1).

This percentage decreased slightly (92.9%) when subjects were asked about the association of SHS exposure and lung cancer in non-smokers. Negative perceptions towards SHS exposure remained consistent through all responses—above 90%. Subjects also expressed their support that public places should be smoke free (95.7%). Table 2 presents the findings of the knowledge and attitude items regarding exposure to SHS. The majority of respondents had good knowledge of the SHS negative health impacts on children. They either agreed or strongly agreed on the proposed negative health effects of SHS exposure on children. This included asthma (89.9%), general child’s health (89.9%), allergies (83.7%), heart attacks (70.8%), low birth weight (85.2%) crib death (Sudden Infant Death Syndrome, SIDS) (68.9%) and ear infections (68.4%). On the other hand, when exploring the participants’ attitudes towards SHS exposure, only 17.7% of women expressed that they will not let their “*visitors*” smoke in their house. However, this percentage became substantially higher as 47.8% of participants expressed that they would ask “*people*” around them to put out their cigarettes.

When summed to create a total score for Knowledge of SHS exposure, the group mean was 24 (SD = 4.0) with a possible range of 6–30 points. The scoring method applied indicates that higher scores describe more accurate or better knowledge about SHS. When summed to create a total score for Attitude about SHS exposure, the group mean was 23.5 (SD = 3.3) with a possible range of 6–30 points. The scoring method applied indicates that higher scores show agreement with policies and procedures to reduce SHS exposure.

### 3.6. SHS Avoidance Practices

Furthermore, the findings of the avoidance subscale (Table 3) revealed that the majority of women (74.1%) will try to distance themselves from smokers to avoid the negative effects of SHS on health by choosing the responses “Almost Always True” or “Usually True” versus the responses “Usually Not True” and “Almost Never True”. On the other hand, the majority of women responded that they would allow people to smoke in their homes (75.1%) and will not leave a group of people if someone starts smoking in the group (79.9%). Although a majority (67.5%) of the participants would let people smoke in their car and would also (65.6%) join their family in a smoking areas, more than half (57.9%) report SHS to be offensive. Finally, the majority of respondents (71.7%) acknowledged that they are routinely associated with people who smoke. When summed to create a total score for the Avoidance of SHS subscale, the group mean was 48.7 (SD = 6.6) with total score means ranging from 25–70 points. The scoring method applied indicates that higher scores describe more avoidance behavior.

The Chi-square test indicated a significant difference between knowledge of the anti-smoking policy at work (yes/no) and avoidance efforts by women *(X*^2^ = 81.778; *p* = 0.02). When comparing the sources of SHS exposure at their homes, the Chi-Square tests showed differences between husband’s smoking status (yes/no) and exposure to SHS (yes/no) *(X*^2^ = 204.0; *p* = 0.0001). This finding indicates that women are more exposed to SHS in the home when they have a smoking spouse. Similarly, women reported exposure to SHS at home when they have a family member who smokes (yes/no) *(X*^2^ = 8.116; *p* = 0.04). Exposure to SHS at home was also associated *(X*^2^ = 44.94; *p* = 0.0001) with household members smoking during the past 30 days (yes/no).

To provide a more comprehensive comparison between subjects in academic positions versus staff or managerial positions, mean scores for Knowledge, Attitudes and Avoidance were compared using Analysis of Variance (ANOVA). The mean scores for the Academic subsample (n = 37) are as follows: Knowledge–24.5 (SD = 3.1); Attitudes–23.5 (SD = 2.0); and Avoidance–47.5 (SD = 5.0). The mean scores for the Staff subsample (n = 173) are as follows: Knowledge–24.0 (SD = 4.0); Attitudes–23.5 (SD = 3.3); and Avoidance–43.5 (SD = 6.6). The ANOVA only showed a significant difference between groups for Avoidance behaviors (F = 11.6, *p* = 0.001).

## 4. Discussion

The present study provides a snapshot of SHS knowledge, attitudes, and avoidance behaviors regarding SHS exposure among educated Jordanian women. The results were generated using a convenience sample of women working at two Jordanian universities. The main rationale for choosing this sector of the population was to test the common belief that educated working women experience less SHS exposure than women with poor education or lower SES as reported by others [11]. Previous research has showed that socioeconomic disparities are directly linked to higher SHS exposure for women and children, partly because of lack of knowledge and partly because of higher smoking prevalence among populations of lower SES [2,7,9]. However, the findings of this study call this commonly held belief into question.

The sample of the study consists of relatively higher SES employed and married women. All of the participants were employed in university settings where policies are present that restrict smoking to designated areas only. However, most respondents were unaware of these policies to address SHS exposure in their workplace. Nearly half of the sample (48%) indicated that there was no policy or they did not know if there was a smoking policy. This lack of knowledge and the apparent lack of enforcement of anti-smoking policies contribute to the women’s extensive SHS exposure. Nearly two-thirds (60%) of participants reported living with a smoking husband and/or other family members who smoke. Almost all respondents reported that their spouses/ family members smoked inside the homes and that smokers consumed an average of 23 cigarettes a day for almost six days a week. An impressive number (71.7%) reported that they routinely associated with people who smoke.

Men typically have higher rates of smoking which leads to higher levels of household SHS exposure among non-smoking women and children [12]. Similar findings have been documented in studies of SHS exposure among Jordanian women and children [6]. The findings presented here are consistent with these earlier studies as well. This study shows that educated Jordanian women can also lack sufficient knowledge, positive attitudes about SHS exposure and then effectively avoid SHS exposure in their private and public lives. Others have documented that women effectively reduce smoking in the home for the benefit of their children. However, their scope of impact is often limited to the household setting [12].

Living with smokers has a negative impact on a non-smoker’s practical ability to avoid to SHS exposure [15]. Likelihood of exposure can be compounded when families are not aware of the danger of exposure, or the adverse health consequences of SHS exposure to children in the household. Jordanian women are no exception. In spite of educational level and knowledge of SHS risks, SHS exposure was prevalent among their children too (92.2%). In addition, most women (74.1%) thought that distancing themselves from smokers within the same area was effective in reducing SHS exposure, but this is not entirely correct. Only 100% smoke free places are sufficiently protective against the hazards of SHS exposure [3].

From a social perspective, Jordanian women are not fully empowered in Jordanian society; the gender gap in families is notable. For example, the World Economic Forum publishes a Global Gender Gap Index; in 2010 Jordan ranked the 120th among 134 countries surveyed [16]. Considering the social and cultural aspects of Jordanian society, it is not surprising that women have difficulty negotiating for smoking restrictions in the home and in the workplace. This is only compounded by the fact that smoking is an addictive substance. Thus, male smokers in Jordan are culturally and physically resistant to smoking restrictions. In lower socio economic classes, this is expected to be prominent as families are often more culturally conservative and more traditional. This is consistent with the finding that SHS exposure was more common in home settings than elsewhere (see Figure 1). Likewise, Hughes and colleagues [17] reported that SHS was encountered by 31% of respondents during a typical day. Among those exposed, the most common exposures occur at work and home.

Jordan prohibits smoking in public places and workplaces, but compliance with the anti-smoking laws is extremely poor in most locations, perhaps because more than half of the Jordanian men smoke. Also, little is known in the general population about the actual dangers of SHS exposure at any given level and the right practices to avoid it [18]. Advocacy for the right interventions to avoid exposure to SHS should be initiated among the Jordanian society as a whole. The benefit from that is twofold, first it would create the awareness on the non-smoker’s predominantly female population and second, it would enhance acceptance and understanding by men. In other words, this would help reverse the social norm about smoking in Jordanian society. Ding found that exposure reduction of SHS can be achieved by changing exposed non-smokers’ behavior through more efficient avoidance practices [19].

These findings support creation of public initiatives to support enforcement of smoking restriction in the workplace and public areas, as well as promoting tobacco-free homes. However, enforcement efforts need to be coupled with smoking cessation programs [20] in order to establish a new smoke-free cultural norm in Jordan. To capitalize on importance of children in Jordan [10], exposure prevention programs should capitalize on adverse maternal-child health effects of SHS exposure. This would promote a smoke-free environment for the benefit of the whole population by both men and women.

## 5. Summary and Policy Implications

### 5.1. Study Limitations

The study used a convenience sample. It was conducted at two public universities; women who work in these universities do not represent all women in Jordan. This limits the generalizability of the study results. The study used a self-report questionnaire where recall bias may have affected responses. There was 30% non-response rate in the study; non-respondents might have held views that differ from those of respondents. These factors might have altered the findings of this study. Moreover, the sample did not include smokers and their views of SHS exposure.

### 5.2. Policy Implications

The results revealed that knowledge of the health impact of SHS exposure did not have a comparable impact on avoidance behavior. Especially among staff and administrative workers, SHS exposure was good but the level of avoidance was low. With the increase in use of email and the Internet in university settings, multimedia messages could be used to reach students and women in the workplace. For women with less education or who are not employed outside the home, a life-skills enhancement approach would be a feasible approach to SHS education and empowerment. In addition, advocacy efforts should target the smoking population (predominantly male) to teach about health hazards and shape attitudes related to SHS exposure. This approach can be crafted to minimize resistance to smoke-free home environments [16]. Focusing on child health is a powerful motivator in the Jordanian culture because children are highly valued.

Since half of these educated working women were uninformed about smoking restriction policies, university administrators need to increase efforts to educate students, faculty and staff using social media, and Internet-based campaigns. Creating a smoke-free environment does not occur overnight; advocacy must be combined with consistent and visible enforcement.

### 5.3. Recommendations for Further Research

More research about SHS knowledge, attitudes and avoidance behaviors are needed in settings such as hospitals, schools, and other institutions to enhance the generalizability of study results. Intervention studies are also needed using large random samples to examine the effect of SHS avoidance programs for the home and especially for poorly educated, low income and women. Future research will be needed to develop and test interventions tailored to meet the needs of various segments of Jordanian society. Adults, teens and children will need special programs to prevent tobacco use, to provide cessation treatment and to reduce SHS exposure.

### 5.4. Conclusions

The level of exposure to SHS is relatively high among well educated Jordanian women. The findings support the need for effective strategies to reduce the prevalence of smoking in Jordan and protect non-smokers from SHS exposure. Moreover, culturally tailored intervention programs will need to be developed to target both smokers and non-smokers of all ages. Smoking is a culturally sensitive issue in Jordan, especially among men. However, due to the overall increase in poor health outcomes due to smoking and SHS in Jordan and the Middle East, it is critical to reverse this harmful social norm that accepts and tolerates smoking.

## Figures and Tables

**Table 1:** Findings from the household SHS exposure scale.

	Disagree (%)	Somewhat Disagree (%)	Somewhat Agree (%)	Agree (%)
Tobacco smoke is dangerous for non-smoker’s health.	2.4	0.00	29.7	67.9
Children who are exposed to tobacco smoke have more illnesses, such as colds.	3.3	3.8	29.7	63.2
Exposure to tobacco smoke can cause lung cancer in non-smokers.	2.4	4.8	32.5	60.3
Public places should be smoke-free.	2.4	1.9	24.4	71.3
Parents or adults should not smoke near children.	6.2	1.9	18.2	73.7

**Table 2:** Findings of knowledge, attitudes, preventive efforts to avoid SHS exposure scale.

	Strongly agree (%)	Agree (%)	Undecided (%)	Disagree (%)	Strongly disagree (%)
**Knowledge**					
Smoke from other people’s cigarettes will shorten my life.	26.8	45.5	13.4	9.1	5.3
Smoke from other people’s cigarettes is harmful for me.	37.8	53.1	5.7	2.4	1.0
Smoking should be banned in all public places.	46.4	46.9	3.3	0.5	2.9
SHS smoke makes my child’s health worse.	41.1	48.8	7.7	1.0	1.4
I let visitors smoke in my home.	16.3	47.4	18.7	8.1	9.6
I ask people around me to put out their cigarettes.	16.7	31.1	30.6	13.9	7.7

**Attitudes**					

SHS causes low birth weight.	35.9	49.3	9.6	3.8	1.4
SHS causes ear infections in children.	22.5	45.9	23.4	6.7	1.4
SHS causes heart attacks to children.	22.0	48.8	22.5	5.3	1.4
SHS is associated with crib death (SIDS).	23.9	45.0	25.8	3.3	1.9
SHS is associated with allergies in children.	35.9	47.8	11.5	2.9	1.9
SHS is associated with asthma in children.	41.1	48.8	6.7	2.4	1.0

**Table 3:** Findings from the avoidance of SHS exposure scale.

	Almost always true (%)	Usually true (%)	Usually not true (%)	Not almost never true (%)
1. When I encounter someone who is smoking, I distance myself to unsure that I will not be exposed to smoke.	26.3	47.8	17.7	8.1
2. I allow people to smoke in my home.	21.5	53.6	15.3	9.6
3. If I am with a group of people, and someone begins to smoke, I will remain with the group.	22.5	57.4	14.4	5.7
4. If I encounter a friend or relative who is smoking, I will sit and talk with him/her while he/she is smoking.	19.6	55.0	19.6	5.7
5. When I am in public place such as restaurant or offices or clinic, I will leave if unable to sit in the nonsmoking section.	13.4	41.1	32.5	12.9
6. When I trip by bus, or any other public transportation I would request a nonsmoking seat.	11.5	23.9	42.1	22.5
7. When I trip by taxi I will ask the driver not to smoke.	14.4	27.3	39.2	19.1
8. I allow people smoking in my car.	13.4	54.1	22.0	10.5
9. If my husband, or friends or relatives are gathering in a designated smoking area to smoke, I will join them rather than be alone.	11.5	54.1	24.4	10.0
10. If I am with people who are smoking and I cannot leave, I will ask them to refine from smoking.	11.5	43.1	32.1	13.4
11. I will sit in the smoking section of an public place or bus station if there are no seats available elsewhere.	13.4	52.2	23.4	11.0
12. When an outdoor functions where smoking is present, I will move a way to avoid it.	14.8	56.5	22.5	6.2
13. When an outdoor functions where water pipe smoking is present, I will move a way to avoid it.	22.5	45.9	25.8	5.7
14. When exposed to secondhand smoke, I wash my clothes solely to remove the smell of smoke from them even if they are otherwise clean.	17.7	40.2	28.7	13.4
15. I find it unpleasant to be around secondhand smoke.	27.3	37.8	19.1	15.8
16. I routinely associate with people who smoke.	23.4	48.3	21.1	7.2
17. When eating out, I always sit in the nonsmoking section.	20.6	46.4	21.1	12.0
18. I frequently places where smoking is prevalent.	13.9	44.5	26.8	14.8
19. I do not find secondhand smoke is offensive.	11.5	30.1	21.1	36.8
